# An Interesting and Rare Case of Hemoglobin D-Punjab Variant in Tamil Nadu

**DOI:** 10.7759/cureus.22668

**Published:** 2022-02-27

**Authors:** Rallapalli Spandana, Karthikeyan Panneerselvam, Sathyamoorthy Mani, Nedunchelian Krishnamoorthy

**Affiliations:** 1 Pediatrics, SRM Medical College Hospital and Research Centre / SRM Institute of Science and Technology, Chennai, IND; 2 Pediatrics, SRM Medical College Hospital and Research Centre, Chennai, IND; 3 Research, Dr. Mehta Multispeciality Hospital, Chennai, IND

**Keywords:** tamil nadu, child, hemoglobinopathy, anemia, hemoglobin d

## Abstract

Hemoglobin D (Hb D), a variant of hemoglobin appears in a few Asian individuals belonging to India, Pakistan, Iran, Iraq, and other parts of the world. In India, it is mainly reported in the North-Western states. Hb D disease causes subclinical jaundice, or it may be asymptomatic with pallor in its heterozygous form. Being a country with hunger and poverty, pallor is always attributed to iron deficiency anemia, but one should not miss out on various other causes.

Here, we report a rare case of Hb D with iron deficiency anemia in a 13-year-old child of South Indian origin (Tamil Nadu) and emphasize the detailed evaluation of cases with severe pallor. He presented with gradually progressing generalized weakness with easy fatigability for the past four weeks. On examination, he was pale, there was no icterus and systemic examination was normal. Investigation revealed a microcytic hypochromic type of anemia. Hb electrophoresis showed the Hb D-Punjab variant. Iron indices were suggestive of iron deficiency anemia. He was advised iron supplements for three months, and he improved with that. He was counseled about his disease and was advised regular follow-up.

## Introduction

Hemoglobin D (Hb D), a variant of hemoglobin appears in a few Asian individuals belonging to India, Pakistan, Iran, Iraq, and other parts of the world [1]. In India, it is mainly reported in the North-Western states. It has not been reported in Tamil Nadu, India. Hb D disease causes subclinical jaundice, or it may be asymptomatic with pallor in its heterozygous form [2]. In India, the prevalence of Iron deficiency anemia in children (6-59 months) is reported as 56% [3], most commonly due to nutritional iron deficiency. India is ranked at the 71st position in the Global Food Security Index, which was released in October 2021. In India, pallor is often associated with iron deficiency anemia, however, various other causes, such as hemoglobin D (Punjab), should not be ignored.

## Case presentation

A 13-year-old well-nourished adolescent boy from Chennai, Tamil Nadu, belonging to the lower-middle socioeconomic status (as per the Modified Kuppuswamy scale), who consumes a mixed Indian diet, presented with severe pallor, easy fatigability, and palpitations for one month. He had a history of pica.

There was no history suggestive of recurrent fever or jaundice, abdominal pain, significant weight loss, tuberculosis, and blood or worms in stools recorded in the past. This boy was a single child, born to fourth-degree consanguineous parents. There was no history of severe pallor or blood transfusion in his parents.

On examination, the child had severe pallor. He was well-oriented. Vital parameters were stable. There was no icterus, edema, lymphadenopathy, or clubbing. Cardiovascular examination showed Grade 3, short systolic murmur (hemic murmur) in the pulmonary area without any thrill. On abdominal examination, there was no organomegaly. Per rectal examination was normal. All other systemic examinations were unremarkable.

Laboratory investigations at the time of admission showed hemoglobin of 4.8 g/dL, serum (S.) iron of 14 mcg/dL, transferrin saturation of 3%, total iron-binding capacity (TIBC) of 483 mcg/dl, S. ferritin of 3.57 ng/dL, and peripheral smear showed microcytic hypochromic anemia. Sickling test, direct and indirect Coombs test, and stool for occult blood were all negative. Renal function tests and USG abdomen and pelvis were normal. 2D echocardiogram was normal.

Since there was severe pallor in a well-nourished adolescent child, the possibility of hemoglobinopathies was also considered, and Hb electrophoresis was done as the initial work-up. Hb electrophoresis revealed Hb F < 0.8%, Hb A0 of 70.3%, HbA2 of 1.8%, and Hb D of 17.4%.

The child was started on oral iron supplements, advised an iron-rich diet at discharge, and was asked to come for review after three months of iron supplementation. The child improved symptomatically with the disappearance of a hemic murmur heard in the pulmonary area. CBC and iron indices on admission and on follow-up revealed the following (Table 1). Peripheral smear was normal post-treatment.

**Table 1:** CBC and iron indices on admission and on follow-up MCHC: mean corpuscular hemoglobin concentration; MCV: mean corpuscular volume; MCH: mean corpuscular hemoglobin; RDW-CV: red blood cell distribution width; nRBC: nucleated red blood cells

	At admission	After 5 months of follow-up treatment
RBC	3.49 millions/cumm	5.42 millions/cumm
Hb	4.8 g/dL	13.3 g/dL
Hematocrit	20.8%	43.5%
MCV	59.6 fl	80.3 fl
MCH	14.3 pg	24.5 pg
MCHC	24 g/dL	30.6 g/dL
RDW-CV	23.9%	14.9%
WBC	9970 cells/cumm	8410 cells/cumm
Neutrophils	70.8%	69.1%
Lymphocytes	24.2%	26.4%
Eosinophils	0.40%	1.80%
Monocytes	3.60%	2%
Basophils	0.50%	0.50%
Immature granulocytes	0.50%	0.20%
nRBC	0.20%	0
Platelets	4.72 lakhs/cumm	3.07 lakhs/cumm

## Discussion

Hb D-Punjab and Hb D-Los Angeles are said to be the same as discovered first in a family of American British multiethnicity, originally of Indian origin, in which glutamine substitutes glutamic acid by position 121 of the β globin chain [4]. Although Hb D is not uncommon in India, its homozygous form is very rare, and very few cases have been reported in the literature [5]. Hb D shows electrophoretic mobility similar to Hb S in alkaline pH, whereas Hb D movement resembles Hb A, in acidic pH [6-7].

A total of 1198 Hb variants have been termed as per the September 2014 Globin Gene Server record. The majority of these Hb D variants do not cause clinical manifestations but some symptomatic cases can have associated hemoglobinopathies. Hb S is the most commonly found trait in the world. Its clinical outcomes are severe when it is in homozygous form or in combination with other hemoglobinopathies like β-Thalassemia, Hb C, and Hb D [8]. Hb DD, the rarest form of inheritance, is usually asymptomatic but few can develop mild or moderate hemolytic anemia [9-10]. Hb D trait cases do not develop hemoglobin D disease or sickle cell disease later in life, but their traits can be passed to their next generations who can have hemoglobin D disease, hemoglobin SD disease, and hemoglobin D/β-thalassemia disease. Hemoglobin D-Punjab, together with sickle hemoglobin, causes severe health issues [11]. Red blood cells in patients with hemoglobin D disease majorly contain hemoglobin D. Excess of Hb D can decrease the RBCs' size and numbers causing mild anemia. Hb D disease generally does not cause serious health problems [11].

When a parent has a Hb D trait while the other parent has a β/0 thalassemia trait, a 25% (1 in 4) risk with every pregnancy is of having a kid with hemoglobin D/β 0 thalassemia (Dβ0) disease, which is a lifetime ailment causing severe health troubles [11]. Molecular studies are always recommended as final investigations but not followed in the majority of the cases.

Pandey S et al. in 2012 showed that hypochromic microcytic cell indices were reported only with clinically asymptomatic Hb D patients for moderate anemia [2]. Dash and colleagues (1988) reported the association between Hb D and hematological malignancies [12].

On searching for Hb D case reports in Tamil Nadu, as well as Hb D and iron-deficiency anemia-related case reports, were not found.

Although the gold standard test for the diagnosis of hemoglobinopathy is a molecular study, most cases are diagnosed with HPLC. A molecular study was not carried out in this index case. In our case, parents were not investigated for hemoglobinopathies.

## Conclusions

Hemoglobin D-Punjab variant is a rare entity seen mostly in the North-Western part of India. But it should also be considered in patients of South India, Tamil Nadu, who are presenting as well-nourished children with severe iron deficiency anemia. This vital differential should always be kept in the mind of physicians, who deal with several alleged hemoglobinopathies.
